# Intimations of Senility

**Published:** 1966-10

**Authors:** T. W. Lloyd


### INTIMATIONS OF SENILITY

BY

T. W. LLOYD, D.M., M.R.C.P.

*Yet though with years my body downward trend*
*As trees beneath their fruit in autumn bend,*
*Spite of my snowy hair and icy veins*
*My head a cheerful temper still retains.*

Blake paints a bearable picture of old age and it is necessary to recognise that while the ageing process inevitably brings change, in the absence of disease normal people should retain understanding and mobility and can expect to continue as acceptable members of society to the end of life.

If ever an organ failed from age alone it would follow that recovery was impossible and progressive further failure must probably ensue with the passage of time. Irretrievable deterioration may be induced by recurrent or progressive disease but this is a different matter and a clear grasp of the distinction is important. Senility with characteristic failing power and changing personality must not be accepted in medical circles as a diagnosis; it is a symptom which demands investigation and explanation.

Geriatric physicians learn this very early. It is not merely that the term senility has a derogatory flavour to which they are hypersensitive, but the misuse of the concept often leads to mismanagement. Once it has been accepted that the patient behaves as he does because he is old there is clearly no more to be done.

The laiety are as much to blame in this matter as are doctors. If it is expected that in late life incontinence of urine will probably occur (and what an offensive idea when one thinks of one's own increasing years), the onset of this symptom, which should call for urgent investigation, excites no comment. A devoted daughter may put up with incontinence of urine for weeks or months when her mother really needs treatment for pyuria or her father slowly passes into uræmia for lack of a prostatectomy. It is sad how often, when at last she does call in medical help, the idea of curing the patient arises less quickly than the idea of disposal. Some examples of this sort of situation follow.

A man of 80, native of another county, was brought to Cheltenham by his landlord and dumped on his brother's doorstep. He had recently developed incontinence of urine but managed fairly well by wrapping himself in towels. Some days later after a tumble he took to his bed and was referred as being " just senile ". On admission to hospital the bladder was found distended, with a suppurating suprapubic stab wound leaking urine. The prostate was large and there were fractures of three ribs. The blood urea was 100 mg%. Some weeks later a transurethral prostatectomy was successfully done and eventually he returned to his native county, an alert and acceptable member of society.

A man of 85 had continued at work till at the age of 84 he developed Parkinsonian symptoms. When he suffered acute retention of urine with a large prostate he was thought unfit for surgery, and was treated by indwelling catheter which was changed at regular intervals. After some months his personality began to change and at last he got a severe mental confusion which was diagnosed as senile dementia. In fact this was a toxic confusional state induced by infection of the renal tract and when this was successfully treated he returned home with full control of his mental faculties and of his bladder.

It is well to remember that there are many possible causes of retention of urine, and a man who has had retention may perhaps not need to persist with his catheter if he can dispense with diuretics. An attempt to manage without a permanent catheter should nearly always be made. The very wearing of this apparatus is enough in some cases to induce depression and personality change.

Another field in which the concept of senility can mislead is that of muscular weakness. As osteoarthritis develops in the elderly it may lead rather to disinclination to use the limbs than to complaint of pain. In such cases if the patient becomes reluctant to move about or actually takes to bed the cause may be missed, and " senile " weakness may be blamed, sometimes with unfortunate consequences. A woman of 89 was referred for this reason. She had been nursed in bed for 18 months by her daughter aged 60 till the daughter's emotional resource was imperilled by the strain. The mother had been well and active till the age of 87 and was mobilised without great difficulty. It seems certain that a wrong opinion had inflicted prolonged hardship on the younger woman.

A similar story sometimes is precipitated by other causes. After months of slowly diminishing mobility during which time he lost the company of his wife, a man of 88 at last took to his bed. His son and daughter-in-law looked after him to the best of their ability but the daughter-in-law found his constant demands for help occasioned by urinary frequency more than she could manage. The general practitioner referred him as being unable to walk and senile, and indeed it required considerable strength for the uninstructed layman to help this man rise from his bed to sit on a commode. The disorder was caused by Parkinsonian rigidity which had developed almost without tremor, and the frequency was due to enlargement of the prostate. Parkinsonian is usually accompanied by obvious tremor, but if this is not present the disorder may be missed, and when rigidity is the principal symptom the results of medicinal treatment are often excellent.

Many old people become unable to walk because they lose their balance and tend to throw their weight behind the heels. If as in such cases as this the muscular strength of the legs is retained, the patient can quite easily be made to stand if the head is brought forward so that the weight of the body is above the foot, not behind the heel. Appreciation of this simple physical fact makes all the difference in helping such patients to move.

Doctors must always be wary of giving bad advice. Having put their patient to bed for excellent reasons, they must put a term to this treatment as soon as possible. A woman of 74 who had been put to bed for some transient ailment was not told to get up. Four years later she was still in bed and the family were worn out for she had grown too heavy to lift ! Such curious stories are not excessively rare.

Malnutrition and deficiency of serious degree is seldom called senility, but minor degrees sometimes are and can easily be treated. Sometimes the patient escapes notice as well as the disease. Often geriatric wards admit cases of anæmia labelled as senility in patients who subsequently become vigorous and independent. Two women, both nearing 80, lived together for 40 years in a state or interdependence in which hatred was as lively a factor as love. The dominant party was admitted to hospital with myocardial infarction and her dependent friend had to come in as well, being " senile ". She was found to have pernicious anæmia, unsuspected because she was not a noticeable person, with a hæmoglobin of less than 5 gm per 100 ml., and when this was treated she became so well able to stand up for herself that the partnership, alas, was soon ruptured.

This is a brief catalogue of patients presenting serious physical or mental impairment of gradual or of relatively sudden onset, which, having been classified as senile (that is by definition irreversible), were found to be due to treatable pathological disorders. There are many more such conditions which lead old people to behave oddly or to abandon the struggle to maintain an active life. These present in endlessly varying patterns and even the doctor who does not readily accept old age as a diagnosis may reluctantly, and perhaps still wrongly, at last embrace it.

A man of 84 lived with his son, the apple of his eye, and his daughter-in-law. For years they dwelt in perfect amity. Gradually the old man became difficult and demanding. Twice he was referred for a surgical opinion because of lower abdominal pain. No organic cause being found his doctor began to fear that the patient was at last showing in his dotage the disapproval he had always felt for his son's wife. Yet all these symptoms were due to Paget's disease of one lumbar vertebra, a very genuine cause of the pain and of his reluctance to stir himself. This diagnosis is well worth making for sometimes radiotherapy can give relief. Still more important is it to recognise spinal osteoporosis in old women, a potent cause of backache and of radiating pain, which should be diagnosed and treated at the earliest possible stage, when the result may be astonishingly good.

The cardiovascular system usually draws attention to its malfunction so definitely that its disorders are seldom attributed to senility, though " senile myocarditis " is a chimera occasionally invoked to explain the symptoms of ischæmic heart disease and myocardial infarction which in old people is often silent. Nor is senility very often diagnosed when changes in health occur rather dramatically. But in the right circumstances it can be easy to overlook the proper explanation of a new event. A woman of 79 took permanently to her bed after an illness diagnosed as pneumonia. Earlier she had been active. She was nursed by a devoted daughter and was put in a nursing home at relatively prohibitive expense from time to time to relieve the household. As funds ran out a new symptom became obtrusive. When she sat out of bed on chair or commode she began with increasing frequency to lose consciousness for up to an hour. This symptom was attributed to her age and her long confinement in bed. It proved however to be due to dramatic postural hypotension due to partial heart block following an infarct which had almost certainly precipitated the whole illness; and this is a condition which can usually be brought under good enough control to permit continued care at home.

Disordered mental function in late years, though usually due to arteriosclerosis, sometimes has a less common cause. Neurosyphilis is still occasionally met in younger patients but it may be diagnosed for the first time at 80, and even then may respond in some degree to treatment. Myxœdema is another cause of mental impairment. The slow narrowing of interest, loss of memory, withdrawal from outside relationships, personality change, and carelessness in a formerly fastidious person may conjure up an impression of progressive senile dementia, but in fact be due to the slow onset of thyroid deficiency. The diagnosis will usually be apparent to the doctor who is on the look-out, but it is easily missed. A particularly good example of this sort of trap was that of a woman of 84 who was referred because she was becoming too much for her daughter to manage. At a domiciliary consultation it was very soon established that there had been no great change in the mother. It was the daughter whose cold, rough handclasp revealed thyroid deficiency, and when she was treated no further complaint was heard of her mother's behaviour.

The first necessity in geriatrics is a profound scepticism as to whether age itself has caused any particular disability. The diagnosis of senility has been admirably described as " the last refuge of the uncritical " and it behoves us always to keep in mind a picture of the normal elderly person. Yet so many people, so many doctors, are ready to look at the age rather than at the individual. To say, as many a house physician has said, " He is 75, he has had a good life " and to feel that nothing more should be done is permissible perhaps if the man is at his last gasp, but if he is destined to live to 90 it is urgently necessary to go to work to reestablish him. Life often remains desirable long after it has ceased to be sweet. In our assessment of the individual his age should be the least considered factor.

